# Aberrances of Cortex Excitability and Connectivity Underlying Motor Deficit in Acute Stroke

**DOI:** 10.1155/2018/1318093

**Published:** 2018-10-21

**Authors:** Juan Du, Jianping Hu, Jingze Hu, Qiang Xu, Qirui Zhang, Ling Liu, Minmin Ma, Gelin Xu, Yong Zhang, Xinfeng Liu, Guangming Lu, Zhiqiang Zhang, Fang Yang

**Affiliations:** ^1^Department of Neurology, Jinling Hospital, Nanjing University School of Medicine, Nanjing 210002, China; ^2^Department of Medical Imaging, Jinling Hospital, Nanjing University School of Medicine, Nanjing 210002, China; ^3^MR Research, GE Healthcare, Shanghai 201203, China; ^4^State Key Laboratory of Analytical Chemistry for Life Science, Nanjing University, Nanjing 210002, China

## Abstract

**Purpose:**

This study was aimed at evaluating the motor cortical excitability and connectivity underlying the neural mechanism of motor deficit in acute stroke by the combination of functional magnetic resonance imaging (fMRI) and electrophysiological measures.

**Methods:**

Twenty-five patients with motor deficit after acute ischemic stroke were involved. General linear model and dynamic causal model analyses were applied to fMRI data for detecting motor-related activation and effective connectivity of the motor cortices. Motor cortical excitability was determined as a resting motor threshold (RMT) of motor evoked potential detected by transcranial magnetic stimulation (TMS). fMRI results were correlated with cortical excitability and upper extremity Fugl-Meyer assessment scores, respectively.

**Results:**

Greater fMRI activation likelihood and motor cortical excitability in the ipsilesional primary motor area (M1) region were associated with better motor performance. During hand movements, the inhibitory connectivity from the contralesional to the ipsilesional M1 was correlated with the degree of motor impairment. Furthermore, ipsilesional motor cortex excitability was correlated with an enhancement of promoting connectivity in ipsilesional M1 or a reduction of interhemispheric inhibition in contralesional M1.

**Conclusions:**

The study suggested that a dysfunction of the ipsilesional M1 and abnormal interhemispheric interactions might underlie the motor disability in acute ischemic stroke. Modifying the excitability of the motor cortex and correcting the abnormal motor network connectivity associated with the motor deficit might be the therapeutic target in early neurorehabilitation for stroke patients.

## 1. Introduction

Stroke is a leading cause of long-term disability worldwide [1]; approximately 80% of acute stroke survivors suffer from persistent different degrees of motor impairment [2]. Research on the neural mechanism of stroke-induced motor impairment is essential for the rehabilitation of the disease. Currently, two related hypotheses have been offered to explain motor impairment of stroke patients. In the first theory, dysfunction of ipsilesional motor areas underlies deficits in motor control after stroke [3, 4], and another posits that transcallosal inhibition (e.g., the contralesional hemisphere exerts a pathologically enhanced inhibitory influences on the ipsilesional hemisphere) thereby contributes to the reduced motor performance of stroke patients [5–7]. In the acute phase of stroke, changes in the brain at different levels from the molecular level to the network level are especially active and accelerate the brain's self-repair processes [8, 9]. Earlier rehabilitation in stroke is more critical for motor functional recovery and has been considered to be associated with better outcomes [10]. Therefore, it is more significant to investigate the neural mechanisms underlying motor deficits in the early stage of stroke, which may provide important theoretical evidence for early neurorehabilitation of ischemic stroke.

Over recent years, multimodality imaging techniques, represented by functional magnetic resource imaging (fMRI), have been widely used for investigating the neural mechanism of the motor deficits in ischemic stroke. By using sophisticated analysis approaches, fMRI has revealed the abnormalities of local activation and connectivity associated with motor deficits after stroke [11]. Dynamic causal modeling (DCM) analysis based on fMRI is a promising approach to capture effective connectivity among key regions of the cortical motor system [12]. As a complementary approach, electrophysiological assessment such as the assessment of the motor evoked potentials (MEPs) by transcranial magnetic stimulation (TMS) provides information about the excitability of the corticomotor system in stroke patients [13]. Nonetheless, these numerous studies used electrophysiological or fMRI measures in isolation. Moreover, most available evidence was primarily limited to the chronic stage after stroke, in which the mechanisms underlying neural reorganization may have returned to being stable [14]. There is little specific information on the characteristics of the motor cortical network in the acute stage following stroke.

Therefore, in the present study, we combined functional MRI and electrophysiological assessments, as well as analyses of effective connectivity to investigate the neural mechanisms underlying motor deficits in acute stroke. We first hypothesized that the early movement-related cortical activation patterns were paralleled by the motor cortical excitability measured by TMS and both relates to the degree of motor impairment after stroke. Then, combining DCM with electrophysiological assessment, we further hypothesized that pathological transcallosal inhibition originating from contralesional M1 to ipsilesional M1 could also occur in the acute stage of stroke and relate to the degree of motor impairment and motor cortical excitability.

## 2. Materials and Methods

### 2.1. Patients

Twenty-five patients (8 females and 17 males; age: 53 ± 10 years) with a first ever ischemic stroke participated in this study. The detailed inclusive criteria included the following: (1) patients being in the acute stage of stroke and having a time from stroke of less than two weeks, (2) single lesion located within the middle cerebral artery territory, as shown by structural MRI, (3) symptoms of motor deficit in the unilateral upper extremity, and (4) absence of aphasia, neglect, apraxia, or epilepsy, which were evaluated according to the patients' clinical symptoms and signs. This study was approved by the Internal Review Board of Jinling Hospital, and written informed consent was obtained from each participant.

### 2.2. Clinical Assessments

The clinical deficit and stroke severity were assessed using the National Institutes of Health Stroke Scale (NIHSS) [15] and the modified Rankin Scale (mRS) [16]. The following motor tests were used to assess the affected upper limb: (1) Fugl-Meyer assessment (FMA) [17] and (2) Medical Research Council (MRC) scale [18] for wrist extension of the affected hand. The FMA is a reliable and standardized motor impairment scale, which ranges from 0 (hemiplegia) to a maximum of 100 points (normal motor performance). It is divided into 66 points for the upper limb (FMA-UL) and 34 points for the lower limb (FMA-LL), scored on a 3-point ordinal scale (0 = cannot perform, 1 = performs partially, and 2 = performs fully). MRC is also used to assess the muscle strength of the hemiplegic side (ranging from 0 to 5; 5 = normal power and 0 = no movement).

### 2.3. Determination of Motor Cortex Excitability

Motor cortex excitability in each hemisphere was measured using single-pulse TMS [19, 20]. TMS was performed using a MagproX100 stimulator (MagVenture Company, Farum, Denmark) with a figure-of-eight coil (outer diameter of one wing 9 cm). Electromyography (EMG) recordings from the bilateral abductor pollicis brevis (APB) were detected using two pairs of silver-silver chloride surface electrodes (Alpine Biomed ApS, Skovlunde, Denmark). The RMT was defined as the minimal stimulus intensity, which could produce a MEP with a peak-to-peak amplitude of at least 50 *μ*V in at least 5 of 10 subsequent trials. The amplitude of MEP to cortical stimulation was measured according to the peak-to-peak voltage (mV) of the EMG response. The latency of MEP was determined as the interval between the onset of the stimulation and the onset of the EMG response (ms). The presence of a TMS-elicited MEP was categorized as present or absent.

### 2.4. fMRI Paradigm

A block-designed motor task, for which the thumb and the forefinger of the affected and unaffected hand had to be tapped together at a rate of 1 Hz, was used as an fMRI activation paradigm. The MRI session consists of five trainings, lasting 5 minutes in total. Each training contained three blocks: a 20-second block of movement of the affected hand, a 20-second block of movement of the unaffected hand, and a 20-second rest block. Two motor task blocks were pseudorandomized and separated by a rest block. Every block was repeated 5 times in total. An investigator carried out the passive movement in the scanner room and ensured the consistency of the motor tasks. All patients underwent motor task training before MRI scanning and performed the passive movements during task-evoked fMRI scanning.

### 2.5. fMRI Data Acquisition

Whole-brain fMRI scans were performed using a GE MR750 3.0 Tesla Scanner (General Electric, USA). We used a gradient echo planar imaging (EPI) sequence with the following imaging parameters: TR = 2000 ms; TE = 30 ms; flip angle = 80°; FOV = 240 mm × 240 mm; matrix = 64 × 64; slice thickness = 3.2 mm, no gap; and slice number = 43 and 160 volumes. The slices covered the whole brain extending from the frontoparietal cortex to lower parts of the cerebellum. We also used 3D-BRAVO sequence to generate high-resolution 3D T1-weighted structural images, with these parameters: TR = 8.2 ms, TE = 3.2 ms, flip angle = 12°, FOV = 220 mm × 20 mm, matrix = 256 × 256, and slice thickness = 1 mm.

### 2.6. MRI Data Preprocess

Considering the effect of stroke lesion on the spatial normalization, a cost function modification was used to remove the presence of a lesion [21]. First, the lesion masks were manually traced on the individual structural 3D T1-weighted images independently by 2 radiologists (Hu JP and Zeng FY), with the guide of the DWI image. Second, a group-sample-specific brain template was generated. For each subject, the lesion-removed whole-brain mask was used as the cost function, to normalize the 3D T1-weighted image into the standard brain template of the Montreal Neurological Institute (MNI) by using a 12-parameter affine transformation with nonlinear adjustments with 78 × 7 basis functions. All individual-normalized 3D T1-weighted anatomical images and all lesion-removed masks were averaged to yield sample-specific brain templates. Third, the averaged 3D T1-weighted anatomical image was segmented using unified segment function of SPM8, with the averaged lesion-removed mask as the cost function. Fourth, the individual space 3D T1-weighted images were segmented using unified segment function. The segment issues of the third step process were used as the templates of the current segment process, and the individual lesion-removed brain mask was used as the cost function. The segment parameters contained the affine transition matrix, which would be used in the functional MRI image normalization [21, 22].

For the functional MRI data, the process was done by DPARSF (http://rfmri.org/DPARSF) and SPM8 (http://www.fil.ion.ucl.ac.uk). Prior to data analysis, images from patients with right-sided lesions were flipped to the left side at the midsagittal plane, so that the affected hemisphere corresponded to the left hemisphere in all patients. First, slice timing and realignment were performed. Translation or rotation parameters in any given data set did not exceed 1.5 mm or 1.5 degree. Second, the functional images were coregistered to the individual 3D T1-weighted images. Third, the segment parameters were applied to the coregistered functional images for the normalization of functional images, with resample of 3 × 3 × 3 mm^3^ voxel size. Fourth, spatial smoothing (FWHM = 8 mm) was performed to the normalized functional images [21, 22].

### 2.7. fMRI Data Analysis

#### 2.7.1. Motor Activation Detection

For each subject, the experimental conditions were modeled in the framework of the general linear model (GLM). The block designs were convolved with a canonical hemodynamic response function applied in SPM8, and the first-order temporal derivatives were also generated. The design matrix also contained the head motion parameters to remove the effects. The contrast maps of the affected and unaffected hand movement were regrouped to calculate the second-level group effect by using SPM8.

#### 2.7.2. Dynamic Causal Modeling

Dynamic causal modeling [23] was used to assess effective connectivity within bilateral cortical motor areas activated by the fMRI motor task. Connectivity parameters represent the connectivity strength from one brain region to another. According to the previous studies [5, 12], six motor regions of interest (ROIs) from the fMRI motor task activation were selected for the DCM analysis: the primary motor area (M1), the premotor cortex (PMC), and the supplementary motor area (SMA) from both hemispheres of each subject (Table 1). A 4 mm radius sphere was applied to the ROI extraction. Principal component analysis was used to extract the ROI that represents signals. Based on previous studies on anatomical and functional connectivity in animals and human beings [4, 5, 12, 24], we supposed the intrinsic connection of these motor areas: connections between SMA and ipsilateral and contralateral M1, between SMA and ipsilateral PMC, and between PMC and ipsilateral M1 and transcallosal connections between M1-M1 and SMA-SMA. Bayes factors were computed for the “winning” model providing the best fit between accuracy and generalizability.

### 2.8. Statistical Analysis

Statistical analyses were performed on SPSS for Windows version 22 (IBM Corp., Armonk, NY). The comparison of neurophysiological measures in the bilateral hemisphere was analyzed by a two-tailed paired *t*-test, with a statistical significance set at *p* < 0.05. The estimated connectivity parameters of the “winning” model in patients were tested for statistical significance by means of one-sample *t*-tests (*p* < 0.05). Pearson correlation analyses were used to test the relationships between fMRI results (neural activity and connectivity parameters) and motor performance, as well as cortical excitability measures. The level of significance was set at *p* < 0.05.

## 3. Results

Table 2 shows the demographic, imaging, and clinical characteristics of these patients.

### 3.1. Cortical Excitability

**Figure 1:**
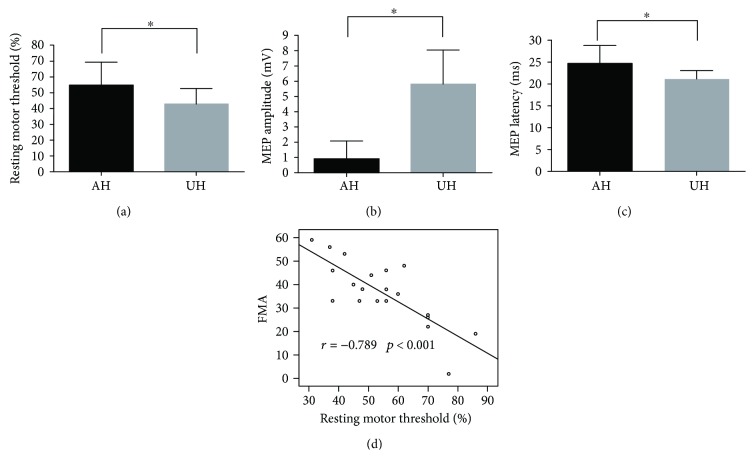
Cortical excitability as a resting motor threshold (a), MEP amplitude (b), and MEP latency (c) of the ipsilesional and contralesional hemisphere in patients. Significantly decreased cortical excitability in the ipsilesional hemisphere compared to the contralesional hemisphere (*p* < 0.05, pair *t*-test). (d) A significant negative correlation was evident between the resting motor threshold of the ipsilesional hemisphere and the FMA score, with reduced cortical excitability in the ipsilesional hemisphere featuring stronger motor impairment. FMA: Fugl-Meyer assessment. ^∗^Significant *P* value < 0.05.

In this study, MEPs could not be elicited in five of the 25 patients that were excluded from neurophysiological data analysis. We found a significant decreased cortical excitability in the ipsilesional hemisphere (increased RMT, reduced MEP amplitude, and prolonged MEP latency) compared to the contralesional hemisphere (*p* < 0.001, *p* < 0.001, and *p* = 0.001, resp., paired *t*-test) (Figures 1(a)–1(c)). The RMT of the ipsilesional hemisphere significantly correlated with FMA scores of the affected limb (*r* = −0.789, *p* < 0.001) (Figure 1(d)), suggesting that better motor performance and less intensity were needed to evoke an MEP in ipsilesional M1. However, no significant correlation was evident between the RMT of the contralesional hemisphere and FMA scores of the affected limb (*r* = −0.229, *p* = 0.331).

### 3.2. fMRI Motor Activation

**Figure 2:**
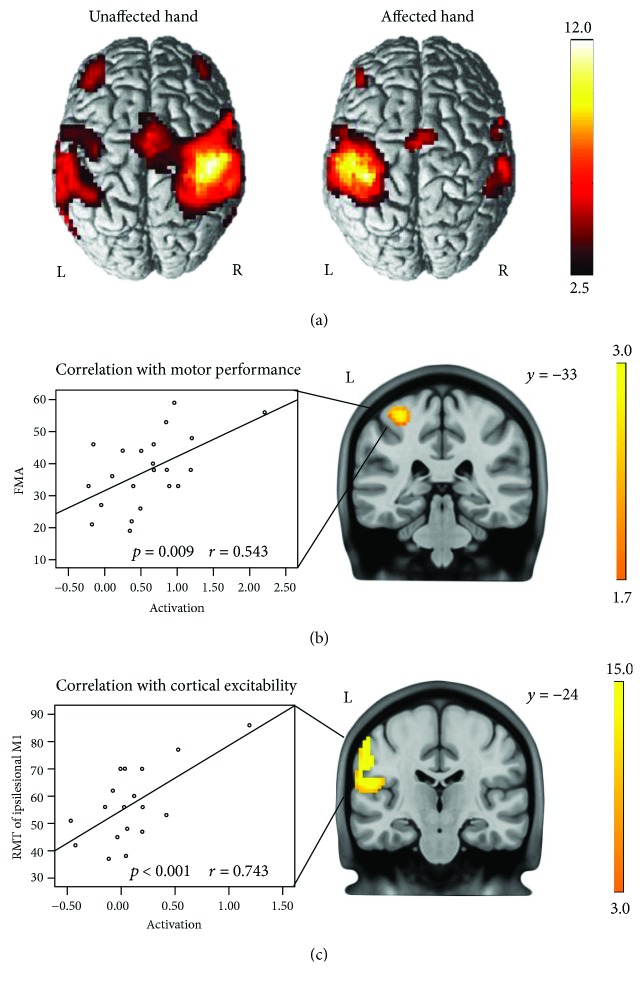
(a) Neural activity during movements of the unaffected and affected hands (*p* < 0.01, FDR corrected). (b) Significant correlation of BOLD activity with FMA of the upper extremity in ipsilesional M1 (*p* < 0.05, uncorrected) for movements of the affected hand. (c) Significant correlation of BOLD activity with the RMT of the ipsilesional hemisphere in the ipsilesional sensorimotor area (*p* < 0.05, uncorrected). L: left; R: right.

We first examined the task-related activation during hand movements. Movements of the unaffected hand were associated with significant BOLD activity in the contralateral M1, SMA, bilateral PMC, and the contralateral somatosensory cortex, consistent with previous studies in healthy subjects [25]. In contrast, although movements of the affected hand revealed BOLD activity in areas homologous to those just mentioned, neural activity decreased in both hemispheres, especially within M1 of the ipsilesional hemisphere (Figure 2(a)).

We next tested for correlations between brain activation early after stroke and motor impairment as well as motor cortical excitability. In the analysis of the relationship between brain activation and motor performance, we ruled out three outliers that were the patients with FMA 2-3 (patients 5, 6, and 12). A significant positive correlation was evident between the levels of BOLD activation within ipsilesional M1 and FMA (*r* = 0.543, *p* = 0.009), with better motor performance in patients featuring higher levels of BOLD activation in ipsilesional M1 during movement of the affected hand (Figure 2(b)). In 20 patients with the presence of MEP, we also found a significant correlation between cortical excitability of ipsilesional M1 and the BOLD activation in the ipsilesional sensorimotor area for movement of the affected hand (*r* = 0.743, *p* < 0.001) (Figure 2(c)). In contrast, the level of the BOLD signal in the contralesional hemisphere did not correlate with motor function as well as with cortical excitability of either hemisphere.

### 3.3. DCM

**Figure 3:**
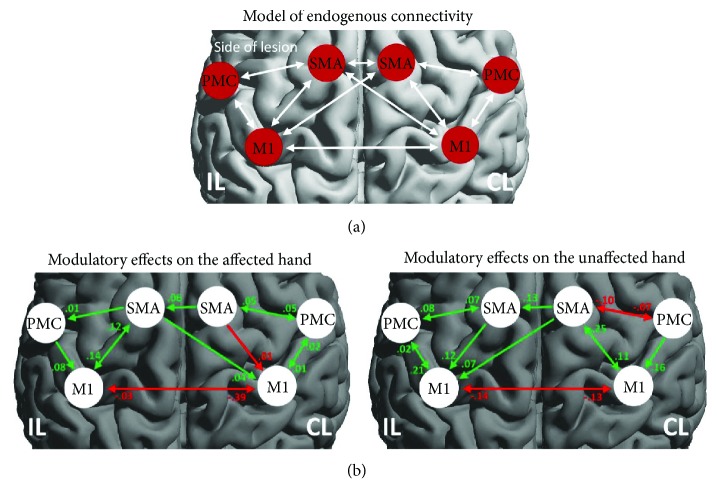
(a) Regions of interest (ROI) and connectivity model used for estimating interregional connectivity. Scans from patients with right-sided lesions were flipped, so that all patients were assumed to have left hemispheric lesions. (b) Task-dependent modulations of connectivity during movements of the affected and unaffected hands. Positive (green) values refer to the promotion of neural activity. Negative (red) values mean the inhibitory influence on neuronal activity. IL: ipsilesional hemisphere; CL: contralesional hemisphere.

Figure 3(a) shows the winning model for endogenous neural coupling. We first analyzed the hand-movement-associated effective connectivity among bilateral motor networks (Figure 3(b)). The interhemispheric connections between ipsilesional and contralesional M1 showed negative connectivity parameters, indicating a mutual suppression of M1-M1 interactions during movement of the affected or unaffected hand (*p* < 0.05, corrected). Furthermore, we also found an additional inhibitory connectivity from contralesional SMA to M1 during movement of the affected hand, as has been described in the previous study [26].

**Figure 4:**
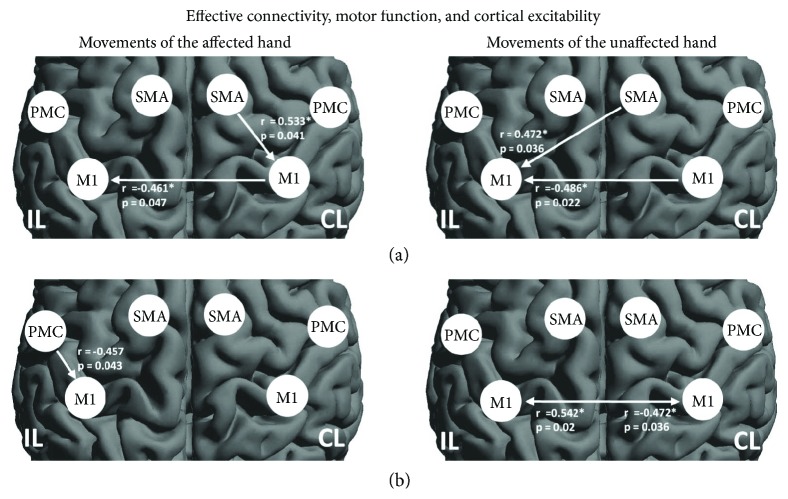
Significant Pearson correlation of DCM connectivity parameters with motor function and cortical excitability. (a) The interhemispheric connections from contralesional-ipsilesional M1 correlated with the degree of motor impairment irrespective of the affected or unaffected hand movements. The motor function also correlated with both contralesional SMA-M1 for the affected hand movement and contralesional SMA-ipsilesional M1 for the unaffected hand movement. (b) Motor excitability of the ipsilesional hemisphere significantly correlated with ipsilesional PMC-M1 during movement of the affected hand and interhemispheric M1-M1 inhibition. IL: ipsilesional hemisphere; CL: contralesional hemisphere.

In the next step, we performed correlation analysis of the connectivity parameters and motor function of the affected upper limb. We found that the FMA correlated negatively with inhibitory connectivity from contralesional M1 to ipsilesional M1 (*p* = 0.047, *r* = −0.461) and positively with inhibitory connectivity from contralesional SMA to M1 (*p* = 0.041, *r* = 0.533) during movements of the affected hand. For movements of the unaffected hand, the FMA correlated with promoting connectivity from contralesional SMA to ipsilesional M1 (*r* = 0.472, *p* = 0.036) as well as with the M1-M1 inhibition (reduced inhibition of ipsilesional M1) (*r* = −0.486, *p* = 0.022, resp.) (Figure 4(a)). These findings were consistent with our fMRI result showing that BOLD activity within the ipsilesional M1 correlated with better motor performance (Figure 2(b)).

Finally, when correlating DCM connectivity parameters with cortical excitability, we found that the stronger the promoting connectivity from ipsilesional PMC to M1 during movement of the affected hand was, the more active the motor cortex excitability was present in the ipsilesional hemisphere (*r* = −0.457, *p* = 0.043; Figure 4(b)). In addition, we also found a statistically significant correlation between the M1-M1 inhibition and RMT of the ipsilesional hemisphere during movement of the unaffected hand (ipsilesional M1-contralesional M1, *r* = −0.472, *p* = 0.036; contralesional M1-ipsilesional M1, *r* = 0.542, *p* = 0.02) (Figure 4(b)). Hence, lower motor cortex excitability (higher intensities are needed to evoke MEP) in the ipsilesional hemisphere featured more intra- or interhemispheric inhibition of ipsilesional M1 and a relative disinhibition of contralesional M1.

## 4. Discussion

Combining neuroimaging and electrophysiological measures, the present study examined the relationships of effective connectivity with motor performance and motor cortical excitability in acute ischemic stroke. We first found that the brain region responding to the affected hand had weaker fMRI activation and cortical excitability relative to those of the unaffected hand. The fMRI activation and cortical excitability in the ipsilesional M1 during movement of the affected hand showed a positive correlation with motor performance. Importantly, DCM analysis revealed both enhancement of positive connectivity in the ipsilesional M1 and negative connectivity in the contralesional M1 correlated with motor performance and the ipsilesional cortical excitability.

### 4.1. Motor Cortical Excitability and Activity

In the present study, the assessments of neural activity and motor cortical excitability provided direct multimodal evidence that the less ipsilesional M1 recruitment signifies poor motor performance in the acute phase after stroke. Our data identified that the reduced cortical excitability and activity in the ipsilesional M1 were associated with the degree of motor impairment in stroke patients. The results were in line with a recent meta-analysis indicating activity in ipsilesional M1 characterizing favorable motor recovery after stroke [27]. Furthermore, we also found a positive correlation between the brain activity in the ipsilesional sensorimotor area and RMT of ipsilesional M1, which implied that the more recruited ipsilesional sensorimotor motor cortex, the less decreased motor cortical excitability of the ipsilesional hemisphere. The parietal lobe of the cortex is responsible for somatosensation and has been shown to be involved in sensorimotor integration for hand coordination [28]. This finding may, at least in part, be related to the fact that passive motor task influenced the neural activity in the somatosensory cortex. However, due to the variance in RMT that was considerably larger in the ipsilesional motor cortex, this observation needs further investigation.

### 4.2. Effective Connectivity in Motor Areas

Importantly, we combined fMRI and neurophysiological measures to investigate the effective connectivity in key motor regions in relation to cortical excitability and motor performance in the acute stage of stroke. Our findings further confirmed the former studies enrolling subacute or chronic stroke patients and demonstrated that a reduction of pathological transcallosal influences (originating from contralesional M1 to ipsilesional M1) may underlie improved motor performance [3, 26].

In the concept of interhemispheric inhibition model theory, both hemispheric M1 exhibit a mutual inhibitory influence on each other [29, 30], which is reflected in the present study data by showing a mutual suppression of M1-M1 connections. We demonstrated that patients with more enhanced inhibitory connectivity to ipsilesional M1 or less intra- and interhemispheric inhibition to contralesional M1 showed stronger motor impairment and lower ipsilesional cortical excitability. In addition, our data demonstrated that the neural coupling between contralesional SMA and contralesional M1, as well as ipsilesional M1, significantly correlated with the degree of motor impairment. More recent neuroimaging and electrophysiological data provided the evidence of the role of SMA showing that a large proportion of SMA neurons exclusively respond to contralateral hand movements only [12, 31]. These findings are compatible with our data suggesting that the SMA might represent a key region promoting or inhibiting the influence on the cortical motor network during movements of the affected or unaffected hand.

### 4.3. Limitation

Several limitations were noteworthy. Firstly, given the cross-sectional design of the study, we can only provide preliminary evidence of correlations across different modalities, without identifying their causative interactions. Future longitudinal studies can be performed to investigate whether the factors identified in the present study (combination of RMT, BOLD activation, and effective connectivity) have a predictive value in the acute poststroke phase for long-term recovery. Secondly, this study did not involve the healthy participants as controls.

## 5. Conclusions

By combining fMRI and electrophysiological measures, this study revealed the aberrances of cortex excitability and connectivity in the acute stage of stroke. The findings contributed to the understanding of the pathophysiology of motor impairment in the early phase of stroke. The inhibitory connectivity to ipsilesional M1 and a relative disinhibition of contralesional M1 may constitute an important pathophysiological aspect of motor disability in acute stroke. Moreover, the present findings might have important implication for early rehabilitative therapy in stroke patients. Aberrances of the cortical excitability and connectivity in the ipsilesional M1 might be considered the therapeutic targets in a few of novel therapeutic strategies (e.g., noninvasive brain stimulation) for the early stage of stroke [32–34].

## Figures and Tables

**Table 1:** Individual fMRI activation maxima used as ROIs for DCM.

	M1_L			M1_R			SMA_L			SAM_R			PMC_L			PMC_R		
Subj1	−39	−24	45	42	−21	54	−12	−18	51	6	−3	57	−51	−3	33	54	−3	21
Subj2	−39	−24	63	33	−27	57	−3	0	72	9	−9	72	−54	−3	42	60	−6	36
Subj3	−39	−21	66	36	−27	69	−6	−3	63	3	−6	69	−51	−3	42	51	−9	33
Subj4	−45	−12	54	36	−21	57	−12	0	66	6	−3	69	−48	0	33	57	−6	42
Subj5	−39	−36	63	39	−21	57	−9	−6	66	12	−9	75	−57	−3	33	60	−9	42
Subj6	−39	−24	66	48	−15	60	−12	3	69	6	−6	66	−42	6	48	60	6	39
Subj7	−36	−30	63	39	−30	63	−6	−6	69	6	−21	69	−54	0	45	51	−3	45
Subj8	−48	−15	54	42	−21	66	−9	9	72	9	−6	72	−54	−3	36	57	0	39
Subj9	−45	−15	48	51	−15	45	−9	6	63	9	6	66	−48	−3	42	60	3	36
Subj10	−48	−15	45	39	−21	57	−9	12	69	6	−6	69	−57	0	30	57	0	42
Subj11	−45	−15	51	45	−15	57	−6	−6	69	9	−12	60	−51	−3	33	60	3	33
Subj12	−39	−21	60	39	−24	54	−6	−12	69	9	6	69	−51	−6	36	51	−3	33
Subj13	−39	−15	57	42	−15	57	−3	9	69	9	6	54	−45	−9	36	57	−6	33
Subj14	−33	−27	54	36	−24	54	−6	−3	69	9	−3	69	−57	0	30	54	0	45
Subj15	−36	−27	66	36	−27	63	−3	−3	69	6	0	66	−54	0	36	60	3	33
Subj16	−39	−21	57	39	−21	57	−6	0	57	9	6	60	−60	3	33	51	−3	39
Subj17	−39	−21	63	48	−15	57	−9	−12	69	6	−6	72	−51	−3	39	51	0	42
Subj18	−39	−24	60	36	−21	54	−3	−9	69	9	6	54	−48	−6	36	60	−3	36
Subj19	−48	−15	54	42	−24	66	−6	−3	69	9	−3	69	−60	0	24	57	0	33
Subj20	−48	−18	51	42	−24	57	−9	−12	66	9	−9	69	−54	−6	36	54	−6	36
Subj21	−36	−27	51	42	−18	57	−6	6	63	6	−3	60	−48	−6	33	57	−9	36
Subj22	−48	−18	45	39	−33	66	−6	3	66	9	−12	72	−54	0	30	51	−3	39
Subj23	−36	−24	51	36	−27	60	−9	−12	69	9	−9	66	−45	−3	45	54	−6	36
Subj24	−39	−18	60	39	−24	57	−6	21	63	9	18	66	−42	−3	45	54	−3	39
Subj25	−45	−15	48	42	−18	60	−6	6	51	6	−12	63	−42	−3	36	51	−9	42

Note: six motor regions of interest (ROIs) from the fMRI motor task activation were selected for the DCM analysis: the primary motor area (M1), the premotor cortex (PMC), and the supplementary motor area (SMA) from both hemispheres of each subject.

**Table 2:** Demographical, imaging, and clinical data of stroke patients.

Patient	Age, y	Sex	Hand dominance	AH	Stroke location	Infarct volume, mL	Lesion age, d	MEP of AH	NIHSS	mRS	BI	FMA	MRC
1	66	F	R	R	CR, BG	3.1	1	Present	7	4	45	33	3
2	68	M	R	R	Temporal parietal region, BG, CR	21.8	2	Present	12	4	25	19	2
3	61	M	R	R	CR	0.5	8	Absent	5	3	65	44	3
4	68	M	R	R	Parietal occipital region	3.7	6	Present	3	2	90	33	2
5	67	F	R	L	Frontoparietal region	8.1	3	Absent	13	4	25	2	1
6	50	F	R	L	BG	4.7	14	Absent	14	4	20	3	1
7	44	M	R	L	CR	0.8	1	Present	6	2	80	53	4
8	40	M	R	L	BG	0.4	2	Present	8	3	70	38	4
9	59	F	R	L	Frontotemporal region	72	2	Present	13	4	30	36	3
10	60	F	R	R	CR	1.2	8	Present	7	4	50	38	2
11	44	M	R	L	BG	2.8	3	Present	7	4	35	46	3
12	56	M	R	R	BG	0.4	6	Present	13	4	25	2	1
13	50	M	R	R	BG, CR	6.3	1	Present	7	4	50	33	3
14	45	F	R	L	BG	6.1	3	present	5	3	60	59	4
15	50	M	R	R	BG	1.8	7	Present	4	2	65	56	4
16	49	M	R	L	BG, CR	2.3	8	Present	11	4	45	46	3
17	36	M	R	L	BG, CR	2.1	13	Present	6	4	50	26	2
18	58	M	R	L	Parietal occipital region, CR	0.5	7	Present	5	3	80	44	3
19	38	M	R	R	BG	2.6	5	Present	5	4	65	27	2
20	47	F	R	R	Frontotemporal region	9.9	6	Present	9	4	40	33	3
21	52	F	R	L	Frontotemporal region	13.5	3	Absent	12	4	35	21	2
22	43	M	R	L	BG	1.2	3	Present	6	3	75	48	4
23	61	M	R	L	BG	4.6	6	Present	9	4	35	40	3
24	39	M	R	L	BG	1.5	3	Absent	7	4	60	38	3
25	66	M	R	R	BG, CR	6.6	2	Present	9	4	45	32	2

M = male; F = female; R = right; L = left; AH = affected hemisphere; UH = unaffected hemisphere; IC = internal capsule; CR = corona radiata; BG = basal ganglia; MEPs = motor evoked potentials; NIHSS = National Institutes of Health Stroke Scale; mRS = modified Rankin Scale; BI = Barthel index; FMA = Fugl-Meyer assessment of upper extremity; MRC = Medical Research Council scale for hand muscles.

## Data Availability

The data used to support the findings of this study are available from the corresponding author upon request.

## Additional Points

*Highlights.* The neural mechanisms of motor deficit in acute stroke remain unclear. This multimodal study demonstrated the relationships of cortical excitability, effective connectivity, and motor impairment following stroke. Our findings provided important theoretical implication for early rehabilitative therapy in stroke patients.
